# Expectation–Maximization-Based Simultaneous Localization and Mapping for Millimeter-Wave Communication Systems

**DOI:** 10.3390/s22186941

**Published:** 2022-09-14

**Authors:** Lu Chen, Zhigang Chen, Zhi Ji

**Affiliations:** Shaanxi Key Laboratory of Deep Space Exploration Intelligent Technology, School of Information and Communications Engineering, Xi’an Jiaotong University, No. 28 West Xianning Road, Xi’an 710049, China

**Keywords:** simultaneous localization and mapping (SLAM), expectation–maximization (EM), millimeter-wave communication systems, angle difference of arrival (ADOA), the stochastic Monte Carlo approximation

## Abstract

In this paper, we proposed a novel expectation–maximization-based simultaneous localization and mapping (SLAM) algorithm for millimeter-wave (mmW) communication systems. By fully exploiting the geometric relationship among the access point (AP) positions, the angle difference of arrival (ADOA) from the APs and the mobile terminal (MT) position, and regarding the MT positions as the latent variable of the AP positions, the proposed algorithm first reformulates the SLAM problem as the maximum likelihood joint estimation over both the AP positions and the MT positions in a latent variable model. Then, it employs a feasible stochastic approximation expectation–maximization (EM) method to estimate the AP positions. Specifically, the stochastic Monte Carlo approximation is employed to obtain the intractable expectation of the MT positions’ posterior probability in the E-step, and the gradient descent-based optimization is used as a viable substitute for estimating the high-dimensional AP positions in the M-step. Further, it estimates the MT positions and constructs the indoor map based on the estimated AP topology. Due to the efficient processing capability of the stochastic approximation EM method and taking full advantage of the abundant spatial information in the crowd-sourcing ADOA data, the proposed method can achieve a better positioning and mapping performance than the existing geometry-based mmW SLAM method, which usually has to compromise between the computation complexity and the estimation performance. The simulation results confirm the effectiveness of the proposed algorithm.

## 1. Introduction

Recently, millimeter-wave (mmW) communications are considered key ingredients to achieve multiple Gbps link rates in 5G-and-beyond networks [1]. Benefiting from the wide bandwidth of the mmW communication system and the small antenna form given by the millimeter wavelength, mmW communication devices can achieve a high resolution in both the path-delay domain and the path-angle domain [2,3]. Moreover, the mmW propagation occurs in quasi-optical propagation pattern and multipath sparsity due to its low scattering effects [4,5]. Therefore, the mmW communication systems can provide great potential for achieving high positioning accuracy due to the multipath sparsity and the high temporal and spatial resolution of the mmW multipath components (MPCs) [3], which also synergizes in turn with mmW communications in fast beamforming to overcome the high attenuation [6,7].

However, the mmW MPCs-based positioning is severely subject to not only the unknown positions of the MPC sources, which are also the potential features characterizing the environment map, including the reflection mirrors of the physical access point (AP) and the scattering points, but also the uncertain data associations of the MPC measurements to the sources [8,9]. As a viable solution addressing the abovementioned challenges, the multipath-based SLAM (simultaneous localization and mapping) technique can detect and localize the scattering points and the reflective flat surfaces represented by virtual APs and jointly estimate the time-varying positions of the mobile agents. The existing mmW SLAM methods include geometry-based methods, the BP (Belief Propagation)-based methods and RFS (Random Finite Set)-based methods as follows.

The geometry-based methods usually assume that the data association of MPC measurements is realized by the full beam training procedure, which is commonly used in mmW communication systems. They gather the MPC measurements at a large number of random epochs, rather than successive epochs, then localize the agent positions as well as the AP (virtual or physical APs) positions by solving the over-determined geometric equations, corresponding to the geometric relationship between such MPC parameters and the positions of the APs and the MT (mobile terminal) [3,8,10]. Hence, these geometry-based methods can be simply applied to the crowd-sourcing data of the MPC measurements at random time slots, whereas they still have to compromise between low complexity and high information utilization because a large number of the MPC measurements are gathered to accumulate enough information. Based on the angle difference of arrival (ADOA), the JADE (Joint Anchor and Device location Estimation) algorithm in [3] decomposes the NP-hard joint estimation of the AP positions and random MT positions into iterations of two successive LS (least-squares) estimations on the AP positions and random MT positions, whereas such a decomposition requires the relaxation of the geometric constraint on the AP positions, random MT positions and the ADOA measurements; thus, it suffers from losing the partial information contained in the ADOA measurements. In addition, the CLAM (communication-driven localization and mapping) method in [11] estimates the locations of the APs (including physical and virtual APs) through solving a minimal number of equations between the ADOA measurements and the AP shape, which are found offline through automatic expression manipulation techniques, then employs an error-resilient version of the ADOA localization algorithm to estimate the MT position. However, this CLAM method does not make full use of the entire relationships among the ADOA measurements due to avoiding the prohibitively high computational complexity, thus also suffering from performance loss.

By regarding the multipath parameters in successive time slots as measurements of an interacting multiple model (IMM), the BP-based SLAM methods usually employ the factor-graph scheme, which decomposes the high-dimensional joint posterior estimation into recursive low-dimensional posterior estimations, to jointly perform a probabilistic data association and sequential Bayesian estimation of the states of an MT and the potential virtual APs characterizing the map [9,12,13,14,15]. Hence, such BP-based SLAM methods can use not only the multipath information but the time evolution of the MPC parameters in an online manner, thus achieving a better SLAM performance with a relatively low complexity. For example, by modeling the mmW SLAM system as a Bayesian framework with the dynamics state and observation functions, the jointly tracking and mapping method in [13] sequentially estimates the positions of the moving MT and the time-invariant potential features by the factor-graph scheme and provides hard decisions regarding the associations of measurements to sources at each epoch. The proposed BP-based algorithm in [9,14] adapts to time-varying system models by jointly inferring the model parameters along with the MT and map-feature states, by representing the time evolution of the IMM parameters as a Markov chain and incorporating the parameters into the factor-graph problem. However, the strict requirements for the IMM statistical prior and the consecutive MPC parameters limit their application on the available crowd-sourcing MPC measurements.

Furthermore, several RFS (Random Finite Set)-based methods have been proposed for the mmW SLAM [8,16,17,18]. These methods model the potential features corresponding to multipaths at successive epochs as a time-varying RFS with uncertain cardinality and state, then recursively update the posterior of the potential features set and the MT state by using the RFS theory, thus realizing the SLAM. However, such RFS-based methods suffer from an extremely high computation complexity due to the high-dimensional set and the rigorous assumption on the multipath statistical model.

In this paper, a novel expectation–maximization (EM)-based SLAM algorithm has been proposed for mmW systems. Similar to the geometry-based SLAM methods, the proposed algorithm assumes that the data association of the MPC measurements is realized by exploiting the characteristics of the MPCs’ AOD and gains in the full beam training procedure and can be readily applied to the available crowd-sourcing MPCs’ measurement data because it does not require the MPC measurements obtained at successive epochs. By regarding the MT positions as the latent variable of the AP positions, the proposed algorithm reformulates the SLAM problem as a maximum likelihood (ML) joint estimation problem of both the AP positions and the MT positions in a latent variable model; thus, it first employs an efficient stochastic approximation EM method to estimate both the AP positions and the MT positions and finally constructs the indoor map based on the estimated AP topology. Due to the efficient processing capability of the stochastic approximation EM method and taking full advantage of the abundant spatial information in the crowd-sourcing ADOA data, the proposed method can achieve a better positioning and mapping performance than the geometry-based mmW SLAM method, which usually has to compromise between the computation complexity and the estimation performance.

The main contributions of this paper are as follows:

(1) By regarding the MT positions as the latent variable of the AP positions, the mmWave MPC ADOA-based SLAM problem is formulated as the ML joint estimation over both the AP positions and the MT positions in a latent variable model, so that the classical EM scheme can be employed to efficiently solve such an NP-hard estimation problem.
(2) The stochastic approximation EM-based SLAM algorithm is developed to achieve both the better performance and the high computational efficiency. Due to the intractable analytical form of the MT position posterior probability, the stochastic Monte Carlo approximation, with a single sample drawn for each random MT position, is adopted in the E-step to approximately compute the expected statistics. In addition, the AP positions are then updated by the gradient descent-based optimization in the M-step, which monotonically increase the likelihood of the MT position samples with low complexity rather than maximizing it.
(3) The simulation results demonstrate that the proposed method can achieve a better performance than the existing geometry-based mmW SLAM method due to the efficient processing capability of the stochastic approximation EM method and taking full advantage of the abundant spatial information in the crowd-sourcing ADOA data.

The rest of this paper is organized as follows. In Section 2, the related SLAM works are briefly investigated. In Section 3, the virtual AP-based system model is described and the multipath ADOA vector is defined. By exploiting the geometric ADOA relationship in the virtual AP-based system, a novel ADOA and EM-based SLAM method is developed to jointly estimate both the AP positions and the MT positions in Section 4. The simulation results are presented in Section 5 to evaluate the performance of the proposed method. Finally, conclusions are drawn in Section 6.

## 2. Related Works

### 2.1. Visual SLAM Methods

Over the past decades, a great deal of research efforts has been devoted to visual SLAM, which is usually based on the information from ubiquitous optical sensors, commonly considered as cameras equipped by robots. The visual SLAM methods usually split the system into tracking tasks and mapping tasks [19,20,21,22] and fulfills them by aligning the extracted sparse features or the dense pixels against the probabilistic models [19,20] or the dense pixel models [21,22]. Although the existing visual SLAM methods have achieved a satisfactory performance and considerable complexity, such visual SLAM methods still suffer from some practical limitations:

(1) Under some situations, such as nights or complex environments with blockages, the clear images of the surrounding environments are not easy to obtain;
(2) Due to the privacy issue, the users may be unwilling to share the image data openly;
(3) In the scenario with a massive number of mobile agents, the cost of extra optical equipment is unaffordable; thus, not all the mobile agents can be equipped with optical sensors.

Meanwhile, the mmW communication technology in 5G-and-beyond networks brings significant advantages to the multipath-assisted SLAM due to their large bandwidth and beamforming capability. This means a higher resolution in the delay and angular domains can be achieved; thus, efficiently resolving and identifying MPCs can provide great potential for achieving a better positioning and mapping accuracy [3]. Hence, the traditional vision-based SLAM methods are limited in multiple practical scenarios, which can be overcome with wireless mmW multipath-based SLAM solutions.

### 2.2. EM SLAM Methods

As a classic and popular ML method with a low computation complexity, the EM scheme has been usually revitalized in different SLAM scenarios, such as in [23,24,25]. Due to different measurements and different scenarios, the SLAM problem is formulated as different system models; thus, the proposed EM SLAM method and the existing EM-SLAM methods in [23,24,25] are still quite different from each other. The main differences between the proposed EM-based SLAM method and the existing EM-SLAM methods in [23,24,25] are listed as follows.

(a) Different system models

In [23,24,25], the SLAM problem is similarly formulated as the hidden Markov models (HMMs) with a finite number of states and observations. Specifically, based on continuous observations of a moving trajectory, the MT positions are formulated as the hidden Markov states and the landmarks’ positions are regarded as the parameters to be estimated, and the EM scheme is employed for such HMMs to estimate both the MT positions and the landmarks’ positions.

However, the SLAM problem in our method is formulated as the hidden-variable models, instead of HMMs, based on crowd-sourcing observations at random positions, rather than continuous observations of a moving trajectory. By regarding the MT positions as the latent variable of the AP positions, the proposed algorithm reformulates the SLAM problem as the ML joint estimation over both the AP positions and the MT positions in a latent variable model.

(b) Different ways of implementing the E-step

Due to adopting the HMM, the EM-SLAM methods in [23,24] employ the sequential Monte Carlo approximation scheme to obtain the expectation of the probability function, while the EM-SLAM method in [25] uses the Extended Kalman Filtering (EKF) scheme to estimate such an expectation recursively.

On the other hand, our proposed method treats the random MT positions as the latent variable, not the hidden Markov variable, and employs the Monte Carlo approximation to obtain the expectation of the probability function in the E-step. Specifically, considering that a large number of random MT positions involve a prohibitively huge computational complexity in the E-step, we draw a single particle from the posterior of the latent variable for each random MT position, rather than a large number of particles for each latent variable in [23,24], then compute the expected sufficient statistics.

In addition, due to different measurements in different SLAM scenarios, the different observation functions are used in our method and the EM-SLAM methods in [23,24,25]. In order to further simplify the computation of the expectation in the E-step and/or the optimization in the M-step, the methods in [23,25] employ a first-order Taylor expansion to approximate the nonlinear observation model, while the proposed method and the method in [24] directly compute the expectation without any approximation on the nonlinear observation model.

(c) Different ways of implementing the M-step

According to the different ways of implementing the E-step, the EM-SLAM methods in [23,24] employ the explicit maximization scheme to estimate the parameters in the M-step, and the EM-SLAM methods in [25] employ the quasi-Newton minimization method.

Our proposed method uses the gradient descent-based optimization for the APs’ positions as a viable substitute for estimating the APs’ positions in the M-step, which is much more computationally efficient. Although the current optimal AP positions estimates are not exactly obtained in each M-step, such gradient descent-based optimization can monotonically increase the log likelihood of the APs’ positions rather than maximizing it.

(d) Different requirements for initial values of the parameters

Because the EM-SLAM methods in [23,25] use a first-order Taylor expansion to approximate the nonlinear observation function, they need an initial value of the parameters to perform the EM iterations, which is also important for their performance. However, the proposed method and the EM-SLAM method in [25] do not have any requirements for the initial values of the parameters, which is more feasible in practical scenarios.

For clarity, the main differences between the proposed method and the existing EM-SLAM methods in [23,24,25] are listed in the following Table 1.

## 3. System Model

**Figure 1:**
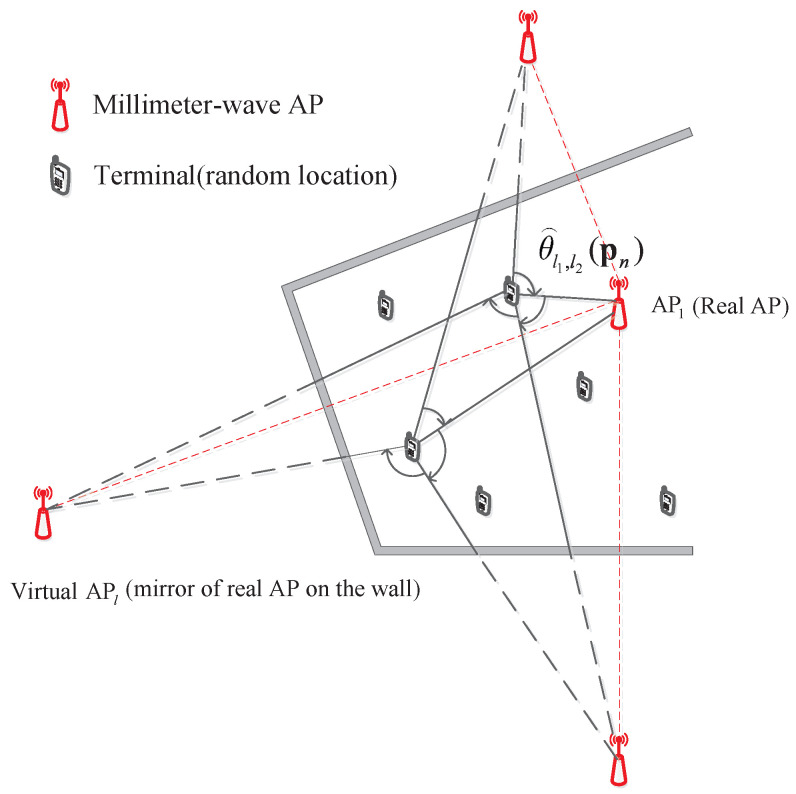
Virtual AP-based mmWave system model.

Consider a two-dimensional indoor scenario (shown in Figure 1) in which a single mmW AP, denoted as ${AP}_{1}$, is deployed and its location $a_{1}$ is unknown. Due to the high attenuation and the quasi-optical propagation of mmW signals in the air [4], only the direct and first-order reflection signal are taken into account as in [3,11] because non-Line-of-Sight (NLOS) paths after higher order of bounces experience enormous attenuation and can be ignored. The first-order reflection NLOS paths can be viewed as the ‘direct’ path from the virtual APs, which are mirrors of first-order reflection of the physical AP through indoor surfaces (such as indoor walls). Denote such virtual APs as ${AP}_{l}\left(l=2,3,\ldots ,L\right)$ with unknown positions $a_{l}\left(l=2,3,\ldots ,L\right)$ and denote the set of all APs as $A=\{AP_{1},AP_{2},\cdots ,AP_{L}\}$, where *L* is the number of APs.

At each position, the MT leverages beam training information, which is available at mmW communication MTs, to compute the angle difference of arrival (ADOA) between the multipath from every AP pair (including the physical AP or virtual APs). Denote the ADOA for the AP pair $\left\{AP_{l_{1}},AP_{l_{2}}\right\}$ at position $p_{n}$ as $\theta_{l_{1},l_{2}}\left(p_{n}\right)$. According to the properties of analytic geometry, the ADOA $\theta_{l_{1},l_{2}}\left(p_{n}\right)$ satisfies:

(1) $$\theta_{l_{1},l_{2}}\left(p_{n}\right)=\arccos\left\{\frac{\left(a_{l_{1}}-p_{n}\right)•\left(a_{l_{2}}-p_{n}\right)}{\left|\left(a_{l_{1}}-p_{n}\right)\right|•\left|\left(a_{l_{2}}-p_{n}\right)\right|}\right\}$$

where • denotes dot-product operation.

From (1), the measured ADOA vector, $\tilde{\theta }\left(p_{n}\right)={\left[{\tilde{\theta }}_{1,2}\left(p_{n}\right),{\tilde{\theta }}_{2,3}\left(p_{n}\right),\cdots ,{\tilde{\theta }}_{L-1,L}\left(p_{n}\right)\right]}^{T}$, at position $p_{n}$ for the AP pair $\left\{AP_{l_{1}},AP_{l_{2}}\right\}$ can be expressed as:

(2) $$\tilde{\theta }\left(p_{n}\right)=\theta \left(p_{n}\right)+W\left(n\right)$$

with

$$\theta \left(p_{n}\right)={\left[\theta_{1,2}\left(p_{n}\right),\theta_{2,3}\left(p_{n}\right),\cdots ,\theta_{L-1,L}\left(p_{n}\right)\right]}^{T}$$

and $W\left(n\right)=\;\;{\left[W_{1,2}\left(n\right),\cdots ,W_{2,3}\left(n\right),\cdots ,W_{L-1,L}\left(n\right)\right]}^{T}$ denoting an additive zero-mean Gaussian noise vector with covariance matrix $\sigma =\mathrm{diag}[\sigma_{1,2}^{2},\;\cdots ,\sigma_{2,3}^{2},\;\cdots ,\sigma_{L-1,L}^{2}\;]$.

Further, we collect the ADOA observation vectors at a large number of random and unknown positions ${\left(p_{n}\right)}_{n=1}^{N}$ and set up a dataset of ADOA vectors as $Q={\left\{\tilde{\theta }\left(p_{n}\right)\right\}}_{n=1}^{N}$. From (1), such defined ADOA vector is inherently immune to orientation biases and completely dependent on positions of the MT and APs; thus, the ADOA vector corresponding to each position is unique and can be collected from different MTs at this position. In addition, the ADOA acquisition only requires the beam training information, which is available and necessary in mmW communication systems. Hence, the setup of such an ADOA observation vector dataset can be handily accomplished in an offline crowd-sourcing manner or even online in mmW communication systems. Specifically, the ADOA observation vectors can be collected or reported from different MTs at random positions whenever they are available in the indoor scenario, rather than from a single MT at only positions on its current trajectory in online manner, and then these ADOA observation vectors can be stored as elements of a dataset in offline manner.

In indoor environments, the main path number or the AP number *L* is usually larger than 3, which implies that the ADOA vector is redundant for estimating the 2-D MT position when the AP positions are known. Hence, both the AP positions and the MT positions can be estimated simultaneously based on collected ADOA vectors at a large number of random positions, because the ADOA vectors contain sufficient redundant information for an extra estimation of the AP positions.

Therefore, the problem of mmW simultaneous localization and mapping for mmW communication systems in an unknown indoor environment, i.e., jointly estimating the geometric topology of APs (including both physical APs and virtual APs) and the MT location, can be reformulated as the following maximum likelihood joint estimation problem:

(3) $$\begin{array}{cc} & \left\{{\hat{a}}_{1:L},{\hat{p}}_{1:N}\right\}\\ & =\arg\max_{a_{1:L},p_{1:N}}𝓅\left({\left\{\tilde{\theta }\left(p_{n}\right)\right\}}_{n=1}^{N}\;|\;a_{3:L},p_{1:N};a_{1:2}\right) \end{array}$$

where 𝓅(·) denotes the probability function, and the semicolon separates the unknown parameters, including virtual sensors’ positions $a_{3:L}$ and the MT positions $p_{1:N}$ to be estimated from the first two APs’ coordinates $a_{1:2}$, which are assumed fixed to resolve the estimation ambiguity of rotation, translation and scaling.

Note that although two-dimension scenarios are assumed in the system model above and the proposed method in the following section, the system model and the proposed method can be extended directly to three-dimension scenarios.

## 4. Algorithm

It is obvious that the estimation problem in (3) is an NP-hard problem considering that both APs’ positions (including both physical APs and virtual APs) and the MT positions are to be jointly estimated. In order to solve this problem, a novel EM (expectation–maximization)-based AP topology estimation method is first proposed in this section. Further, based on the estimated AP topology and the measured ADOA vectors, a least-square-based terminal position estimation method is developed.

Given the observed ADOA measurements at a large number of random positions, $\{\theta \left(p_{n}\right){\}}_{n=1}^{N}$, the APs’ positions $a_{1:L}$ and the MT positions $p_{1:N}$ depend on each other, i.e., the MT positions $p_{1:N}$ can be viewed as the latent variable of the APs’ positions $a_{1:L}$ in (3). Hence, APs’ positions estimation problem in (3) can be equivalently expressed as

(4) $$\begin{array}{cc} & {\hat{a}}_{3:L}=\arg\max_{a_{3:L}}𝓅\left({\left\{\tilde{\theta }\left(p_{n}\right)\right\}}_{n=1}^{N}\;|\;a_{3:L};a_{1:2}\right)\\ & =\arg\max_{a_{3:L}}\left\{\sum_{p_{1:N}}𝓅\left({\left\{\tilde{\theta }\left(p_{n}\right)\right\}}_{n=1}^{N},p_{1:N}|a_{3:L};a_{1:2}\right)\right\}. \end{array}$$

The abovementioned probability maximization problem of the latent variable model in (4) can be efficiently solved by employing the classical EM method [26]; thus, the original ML joint estimation in (3) can be decomposed into 2 feasible steps: (1) estimating the APs’ positions $a_{3:L}$ through the EM method, (2) estimating the MT’s positions based on the estimated physical and virtual APs’ positions. The EM-based virtual sensors’ positions estimation is first discussed hereinafter.

### 4.1. EM-Based AP Topology Estimation

Considering the EM-based virtual sensors’ positions estimation is composed of $T^{\prime }$ similar iterative steps, only the *t*-th iteration is exemplified as follows.

E-Step (Estimation Step):

Given the virtual sensors’ positions estimate ${\hat{a}}_{3:L}^{t-1}$ in the (t−1)-th iteration, the distribution of the MT’s positions $p_{1:N}$ at the *t*-th iteration, ${𝓆}^{t}\left(p_{1:N}\right)$, can be chosen as

(5) $${𝓆}^{t}\left(p_{1:N}\right)=𝓅\left(p_{1:N}\;|\;{\left\{\tilde{\theta }\left(p_{n}\right)\right\}}_{n=1}^{N},{\hat{a}}_{3:L}^{t-1};a_{1:2}\right)$$

Then, the expectation of the probability function $𝓅\left({\left\{\tilde{\theta }\left(p_{n}\right)\right\}}_{n=1}^{N},p_{1:N}|a_{3:L};a_{1:2}\right)$ with respect to the random MT’s positions $p_{1:N}$ can be derived as

(6) $$\begin{array}{c} Q\left(a_{3:L},\;{𝓆}^{t}\left(p_{1:N}\right)\right))\\ =E_{{𝓆}^{t}}\left[𝓅\left({\left\{\tilde{\theta }\left(p_{n}\right)\right\}}_{n=1}^{N},p_{1:N}|a_{3:L};a_{1:2}\right)\right] \end{array}$$

where $E_{{𝓆}^{t}}\left[\cdot \right]$ denotes the expectation operation with respect to the distribution ${𝓆}^{t}\left(p_{1:N}\right)$.

M-Step (Maximization Step):

The APs’ positions estimate ${\hat{a}}_{3:L}^{t}$ at the *t*-th iteration can be obtained by maximizing the expectation function $Q\left(a_{3:L},\;{𝓆}^{t}\left(p_{1:N}\right)\right)$ in (6) as

(7) $$\begin{array}{cc} & {\hat{a}}_{3:L}^{t}=\arg\max_{a_{3:L}}\left\{Q\left(a_{3:L},\;{𝓆}^{t}\left(p_{1:N}\right)\right)\right\}\\ & =\arg\max_{a_{3:L}}\left\{E_{{𝓆}^{t}}\left[𝓅\left({\left\{\tilde{\theta }\left(p_{n}\right)\right\}}_{n=1}^{N},p_{1:N}|a_{3:L};a_{1:2}\right)\right]\right\} \end{array}$$

### 4.2. Stochastic Approximation EM

Because the relationship between ${\left\{\tilde{\theta }\left(p_{n}\right)\right\}}_{n=1}^{N}$ and $a_{1:L}$, $p_{1:N}$ in (1) is complex and nonlinear, the analytical form of the expectation of the probability function $Q\left(a_{3:L},\;{𝓆}^{t}\left(p_{1:N}\right)\right))$ in (6) is generally intractable, thus rendering the analytical approach of the abovementioned EM-based AP topology estimation infeasible. As an alternative way, the stochastic Monte Carlo approximation can be employed to obtain the expectation of the probability function $𝓅\left({\left\{\tilde{\theta }\left(p_{n}\right)\right\}}_{n=1}^{N},p_{1:N}|a_{3:L};a_{1:2}\right)$ in the E-step above [27]. Specifically, considering that a large number of random MT positions involve prohibitively huge computational complexity in the E-step, we draw a single sample from the posterior, ${𝓆}^{t}\left(p_{n}\right)$, for each random MT position $p_{n}$ and then compute the expected sufficient statistics $Q\left(a_{3:L},\;{𝓆}^{t}\left(p_{1:N}\right)\right))$.

By adopting the stochastic Monte Carlo approximation above, the *t*-th iteration of EM-based AP positioning method can be modified as follows.

#### 4.2.1. Modified E-Step

Given the virtual AP position estimates ${\hat{a}}_{3:L}^{t-1}$ in the (t−1)-th step, select *M* MT positions ${\left\{{\bar{p}}_{m}\right\}}_{m=1}^{M}$ randomly in the possible range, then generate *M* corresponding ADOA vectors at such *M* MT positions, according to the properties of analytic geometry:

(8) $$\begin{array}{cc} & \theta \left({\bar{p}}_{m}\right)=\left[\theta_{1,2}\left({\bar{p}}_{m}\right)\right),\theta_{2,3}\left({\bar{p}}_{m}\right)),\cdots ,\theta_{L-1,L}\left({\bar{p}}_{m}\right))]\\ & \;\;\;\;\;\;m=1,\cdots ,M \end{array}$$

with

(9) $$\theta_{l_{1},l_{2}}\left({\bar{p}}_{m}\right)=\arccos\left\{\frac{\left({\hat{a}}_{l_{1}}^{t-1}-{\bar{p}}_{m}\right)•\left({\hat{a}}_{l_{2}}^{t-1}-{\bar{p}}_{m}\right)}{\left|\left({\hat{a}}_{l_{1}}^{t-1}-{\bar{p}}_{m}\right)\right|•\left|\left({\hat{a}}_{l_{2}}^{t-1}-{\bar{p}}_{m}\right)\right|}\right\}$$

On the other hand, the chosen distribution of the MT’s positions $p_{1:N}$ at the *t*-th iteration, ${𝓆}^{t}\left(p_{1:N}\right)$, satisfies

(10) $$\begin{array}{cc} {𝓆}^{t}\left(p_{n}\right) & =𝓅\left(p_{n}\;|\;\left\{\tilde{\theta }\left(p_{n}\right)\right\},{\hat{a}}_{3:L}^{t-1};a_{1:2}\right)\\ & \propto 𝓅\left(\left\{\tilde{\theta }\left(p_{n}\right)\right\}\;|\;p_{n},{\hat{a}}_{3:L}^{t-1};a_{1:2}\right)\cdot 𝓅\left(p_{n}\right)\\ & \propto \prod_{l_{1},l_{2}}N\left({\tilde{\theta }}_{l_{1},l_{2}}\left(p_{n}\right),\sigma_{l_{1},l_{2}}^{2}\right)\\ & \propto \left(\prod_{l_{1},l_{2}}\frac{1}{\sqrt{2\pi }\sigma_{l_{1},l_{2}}}\right)e^{-\sum_{l_{1},l_{2}}\frac{{\left({\tilde{\theta }}_{l_{1},l_{2}}-\theta_{l_{1},l_{2}}\left(p_{n}\right)\right)}^{2}}{2\sigma_{l_{1},l_{2}}^{2}}} \end{array}$$

where the second proportion holds because the ADOA measurement errors for different AP pairs follow the independent normal distributions, i.e., $\theta_{l_{1},l_{2}}\left(p_{n}\right)∼N\left({\tilde{\theta }}_{l_{1},l_{2}}\left(p_{n}\right),\sigma_{l_{1},l_{2}}^{2}\right)$.

Define the weighted Euclidean distance between the observed ADOA vector $\tilde{\theta }\left(p_{n}\right)$ and the generated ADOA vector $\theta ({\bar{p}}_{m})$ as

(11) $$\begin{array}{cc} 𝒹(p_{n},{\bar{p}}_{m}) & =\sqrt{\sum_{l_{1},l_{2}}\frac{{\left(\tilde{\theta }{\left(p_{n}\right)}_{l_{1},l_{2}}-\theta_{l_{1},l_{2}}\left({\bar{p}}_{m}\right)\right)}^{2}}{\sigma_{l_{1},l_{2}}^{2}}}\\ & n=1,\cdots ,N;\;\;m=1,\cdots ,M \end{array}$$

For each observed ADOA vector $\left\{\tilde{\theta }\left(p_{n}\right)\right\}$, the generated ADOA vector with the minimum weighted Euclidean distance can be obtained as

(12) $${\hat{p}}_{n}^{t}=\underset{{\bar{p}}_{m}}{\arg\min}𝒹(p_{n},{\bar{p}}_{m}),\;\;\;n=1,\cdots ,N;$$

By integrating (10)–(12), it can be derived that ${\hat{p}}_{n}^{t}$ is the position estimate for the observed ADOA vector $\left\{\tilde{\theta }\left(p_{n}\right)\right\}$ with the maximum posterior probability among the randomly chosen positions ${\left\{{\bar{p}}_{m}\right\}}_{m=1}^{M}$. Without losing generality, ${\hat{p}}_{n}^{t}$ can be regarded as the single-position sample from the posterior ${𝓆}^{t}\left(p_{n}\right)$ at the *t*-th stochastic EM iteration.

#### 4.2.2. Modified M-Step (Gradient Descent-Based Optimization)

Based on the drawn position samples ${\left\{{\hat{p}}_{n}^{t}\right\}}_{n=1}^{N}$ for observed ADOA vectors, the APs’ position estimates at the *t*-th stochastic EM iteration can be theoretically obtained as

(13) $$\begin{array}{cc} & {\hat{a}}_{3:L}^{t}=\arg\max_{a_{3:L}}\left\{E_{{𝓆}^{t}}\left[𝓅\left({\left\{\tilde{\theta }\left(p_{n}\right)\right\}}_{n=1}^{N},p_{1:N}|a_{3:L};a_{1:2}\right)\right]\right\}\\ & \approx \arg\max_{a_{3:L}}\left\{𝓅\left({\left\{\tilde{\theta }\left(p_{n}\right)\right\}}_{n=1}^{N},{\hat{p}}_{1:N}^{t}|a_{3:L};a_{1:2}\right)\right\}\\ & \approx \arg\max_{a_{3:L}}\left\{𝓅\left({\left\{\tilde{\theta }\left(p_{n}\right)\right\}}_{n=1}^{N}\;|\;{\hat{p}}_{1:N}^{t},a_{3:L};a_{1:2}\right)\right\}\\ & \approx \arg\min_{a_{3:L}}\left\{\sum_{n=1}^{N}\underset{E_{n}^{t}}{\underset{︸}{\sum_{l_{1},l_{2}}\frac{{\left(\tilde{\theta }{\left(p_{n}\right)}_{l_{1},l_{2}}-\theta_{l_{1},l_{2}}\left({\hat{p}}_{n}^{t},a_{3:L}\right)\right)}^{2}}{\sigma_{l_{1},l_{2}}^{2}}}}\right\} \end{array}$$

where the third approximation is obtained by substituting (10) into the second approximation above.

Because the APs’ positions estimate in (13) is still not a closed-form solution and the exhaustive search method for high-dimensional parameters involves huge computational complexity, the gradient descent-based optimization for the APs’ positions is used as a viable substitute for estimating the APs’ positions in the M-step, which is much more computationally efficient. Although the current optimal AP positions estimates are not exactly obtained in each M-step, such gradient descent-based optimization can monotonically increase the log likelihood of the APs’ positions rather than maximizing it.

Hence, the position of the *l*-th AP in the *t*-th iteration is updated as

(14) $${\hat{a}}_{l}^{t}={\hat{a}}_{l}^{\left(t-1\right)}-\alpha \sum_{n=1}^{N}\frac{\partial E_{n}^{t}}{\partial a_{l}},\;\;\;l=3,\cdots ,L$$

where α denotes the learning rate, and the closed-form expression of the gradient, $\frac{\partial E_{n}^{t}}{\partial a_{l}}$, is derived in Appendix A.

The pseudocode for the proposed stochastic approximation EM-based AP positioning method is given in Algorithm 1.

**Algorithm 1:** Stochastic Approximation EM-based AP positioning.**Input:** Observed ADOA vectors ${\left\{\tilde{\theta }\left(p_{n}\right)\right\}}_{n=1}^{N}$ at a large number of random and unknown positions 1:Initialization: ${\hat{a}}_{3:L}^{0},\;{\hat{a}}_{1}=\left[0\;\;0\right],{\hat{a}}_{2}=\left[1\;\;0\right],t=0$2:** repeat**3:   t←t+1    **E-step in *t*-th iteration:**4:   Select *M* MT positions ${\left\{{\bar{p}}_{m}\right\}}_{m=1}^{M}$ randomly5:   Generate $\theta ({\hat{a}}_{3:L}^{t-1},{\bar{p}}_{m})$ based on Equation (8)6:   Obtain the position samples ${\left\{{\hat{p}}_{n}^{t}\right\}}_{n=1}^{N}$ for observed ADOA vectors ${\left\{\tilde{\theta }\left(p_{n}\right)\right\}}_{n=1}^{N}$ according to Equation (12)  **M-step in *t*-th iteration:**7:   update ${\hat{a}}_{3:L}^{t}$ based on ${\left\{{\hat{p}}_{n}^{t}\right\}}_{n=1}^{N}$ according to Equation (14)8:**until**∑l=3La^lt−a^lt−12a^3:Lt2<ε or t>MaxIT **Output:** the AP position estimates ${\hat{a}}_{3:L}^{t}$ and ${\hat{a}}_{1:2}$

### 4.3. Localization of the MT and Construction of the Environment Map

Based on the estimated APs’ positions above, the classical AOA-based positioning method, such as the constrained least-square approach in [10], can be employed to estimate the MT position for each ADOA vector. Further, given by the estimated MT positions ${\left\{{\hat{p}}_{n}\right\}}_{n=1}^{N}$ and the estimated AP positions ${\left\{{\hat{a}}_{l}\right\}}_{l=1}^{L}$, the wall positions can be geometrically calculated as in [11]. Specifically, for each estimated MT position $\left\{{\hat{p}}_{n}\right\}$ and each AP pair at $\left\{{\hat{a}}_{1},{\hat{a}}_{l}\right\}$, the corresponding reflection point on an indoor wall can be estimated as the intersection between the segment $\bar{{\hat{p}}_{n}{\hat{a}}_{l}}$ and the line that bisects segment $\bar{{\hat{a}}_{1}{\hat{a}}_{l}}$. Thus, the indoor wall positions, i.e., the environment map, can be constructed.

## 5. Simulation Results

**Figure 2:**
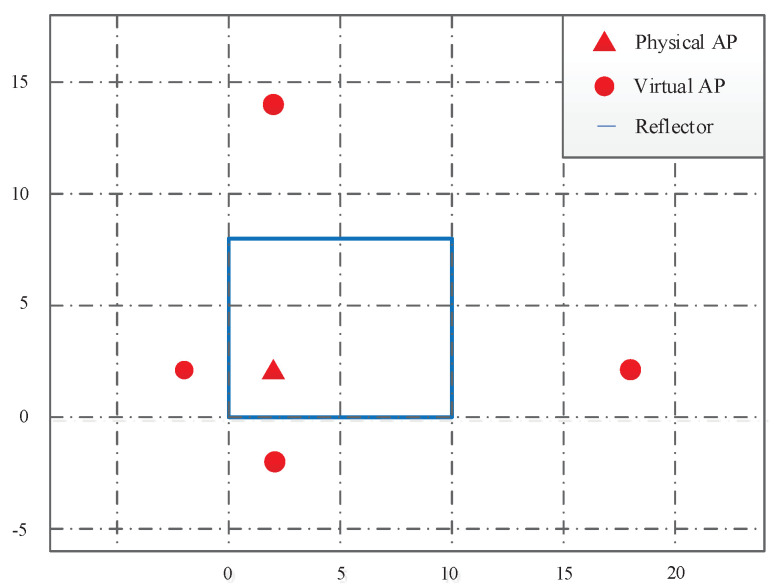
The two-dimension layout of the indoor mmW WLAN environment.

For evaluation purposes, we have conducted the computer simulations in a relatively simple 10 m × 8 m (length × width) two-dimension indoor WLAN environment, shown in Figure 2, in which there is only one physical AP located at (2,2) and four reflective walls. Because the NLOS paths after the higher order of bounces experience enormous attenuation and can be ignored due to the high attenuation and the quasi-optical propagation pattern of the mmW signals in the air [4], only the indirect paths created by a single specular reflection off of a side wall and the direct path are taken into account in the simulations. The four virtual APs are located at the four mirroring positions of the physical AP through the corresponding reflecting walls.

In the simulations, the image-based ray-tracing method is adopted to generate the multipath AOAs at random indoor positions. To derive realistic estimation errors of the AOA measurements, we synthesize the beam shapes for a uniform linear antenna array with 32, 16 and 8 elements. Under typical signal-to-noise ratio conditions, these correspond to the zero-mean Gaussian distributed error of standard deviation $\left\{1^{\circ },2^{\circ },5^{\circ }\right\}$ as in [3,7], respectively. Thus, the ADOA measurement noise is also assumed to follow the zero-mean Gaussian distribution. In addition, the learning rate of the gradient descent-based optimization, the predefined maximum iteration number and the convergence judgment threshold are, respectively, chosen as α=0.02, ε=0.0001 and MaxIT=200, the number of the measured ADOA vectors at random indoor positions is set to 500 and the standard deviation of the AOA measurement noise is set to $2^{\circ }$, which will be kept unless otherwise stated.

For a fair comparison, we compare the localization and mapping performance of the proposed EM-based SLAM method with that of the JADE algorithm in [3] in our simulations, because the JADE algorithm in [3] also requires zero-initial information and can simultaneously estimate the AP positions and the MT positions based on the multipath ADOA measurements in indoor environments with only one AP deployed. In addition, the Cramer–Rao lower bound of localization is simulated as the ideal performance benchmark for the mmW positioning.

### 5.1. Complexity Analysis

In our proposed method, each iteration requires one E-step and one M-step. For the E-step, the main operation includes generating $\theta ({\bar{p}}_{m})$ for M=500 position particles ${\left\{{\bar{p}}_{m}\right\}}_{m=1}^{M}$ based on (8) and computing the weighted Euclidean distance between the observed ADOA vector $\tilde{\theta }\left(p_{n}\right)$ and the generated ADOA vector $\theta ({\bar{p}}_{m})$, according to (12), to obtain the single-position sample ${\hat{p}}_{n}$ for the observed ADOA vector $\tilde{\theta }\left(p_{n}\right)$. Hence, the complexity of our E-step is O(MN+M). For the M-step, the main complexity lies in the calculation of the Q-functions gradient with respect to the parameters, $a_{3:L}$, which is a sum of all individual gradients for each AP and expressed in (14) and (A2). The complexity of each M-step is O(LN). In summary, the proposed EM-SLAM method has a complexity of $O\left(T_{EM}\left(MN+M+LN\right)\right)\approx O\left(T_{EM}\left(M+L\right)N\right)$, where $T_{EM}$ denotes the number of iterations and $T_{EM}⩽200$ suffices for the proposed method to converge.

On the other hand, the JADE algorithm mainly includes the initial estimation of the AP locations and iterative optimizations. The complexity of the initial estimation of the anchor locations is of $O(L^{2}G)$, where $G=2^{16}$ is the number of grid search points for each AP in [3]. By relaxing the geometric constraint on the AP positions, the MT positions and the ADOA measurements, the JADE algorithm transforms the NP-hard joint estimation over the AP positions and random terminal positions into iterative optimization steps; each iteration has a complexity of $O(L^{2}N)$ [3]. As a result, the JADE algorithm has a complexity of $O(L^{2}\left(NT_{JADE}+G\right))$, where $T_{JADE}$ denotes the number of iterations and $T_{JADE}⩽200$ suffices for JADE to converge.

Obviously, both the proposed EM-based SLAM method and the JADE method have the complexity of the same order, which is proportional to the number of measurements. Therefore, the proposed EM-based SLAM method does not require relaxing the geometric constraints of the AP positions and achieves the comparable computation complexity with the JADE method.

### 5.2. Comparison of Localization Performance

**Figure 3:**
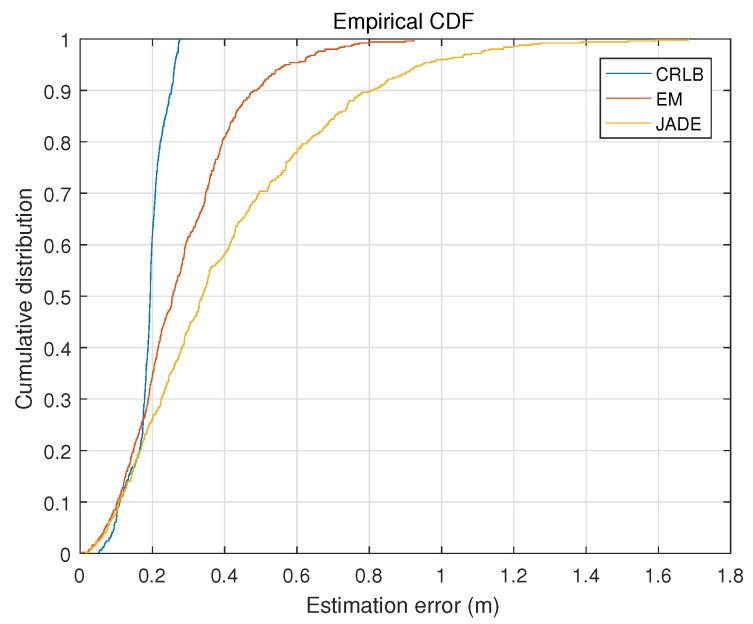
The cumulative probability–distribution curves of localization error.

Figure 3 shows the cumulative probability–distribution curves of the positioning error of the proposed algorithm, the JADE algorithm and the Cramer–Rao lower bound. By relaxing the geometric constraint on the AP positions, the MT positions and the ADOA measurements, the JADE algorithm transforms the NP-hard joint estimation over the AP positions and random terminal positions into iteratively solving two successive LS (least-squares) estimation problems; thus, it suffers from losing the partial information contained in the ADOA measurements. On the other hand, the proposed algorithm formulates the complex SLAM problem as the parameter estimation in a latent variable model and employs the stochastic approximation EM scheme to estimate the terminal position and the APs’ positions without requiring any relaxation of the geometric constraint; thus, it can extract more information from the measured ADOA data than the JADE algorithm. This explains that the proposed algorithm achieves a better positioning performance than the JADE algorithm in Figure 3. Moreover, Figure 3 shows that there is a considerable gap between the cumulative probability–distribution curves of our algorithm and the Cramer–Rao lower bound due to insufficient ADOA vector measurements.

**Figure 4:**
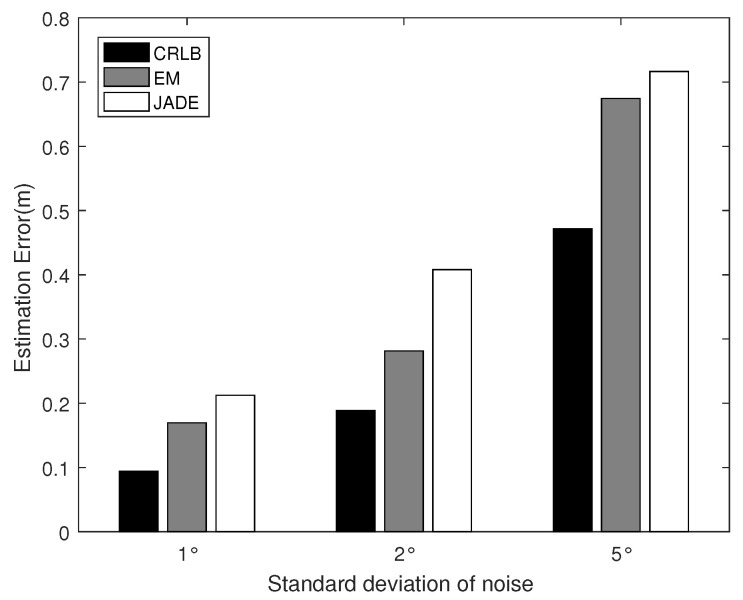
Localization performance versus AOA measurement noise.

**Figure 5:**
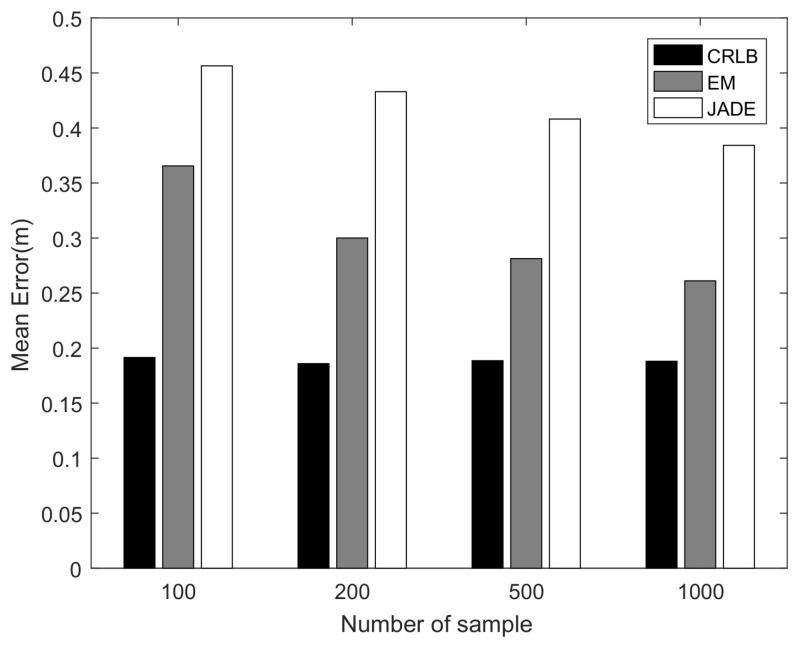
Localization performance versus the number of measured ADOA vectors.

Figure 4 and Figure 5 demonstrate the average positioning error against the ADOA measurement noise and the number of ADOA vector measurements, respectively. From Figure 4 and Figure 5, it is obvious that both the proposed algorithm and the JADE algorithm perform better with the decrease in the standard deviation of the ADOA measurement noise and the increase in the number of samples due to the averaging effects over the more accurate or more multipath ADOA measurements. It also shows that the localization error of the proposed algorithm is lower than that of the JADE algorithm, because the proposed algorithm not only achieves a higher utilization of the information contained in the ADOA measurement data but also works reliably against the measurement noise due to the robustness of the EM algorithm, while the JADE algorithm involves the LS estimation, which is sensitive to the noise.

### 5.3. Comparison of Mapping Performance

**Figure 6:**
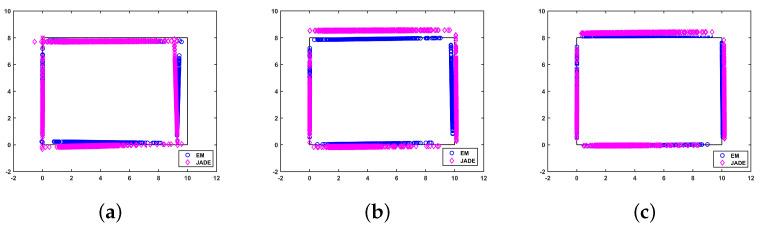
The mapping performance for different standard deviations of AOA errors: N=500. (**a**) $\sigma =5^{\circ }$; (**b**) $\sigma =2^{\circ }$; (**c**) $\sigma =1^{\circ }$.

**Figure 7:**
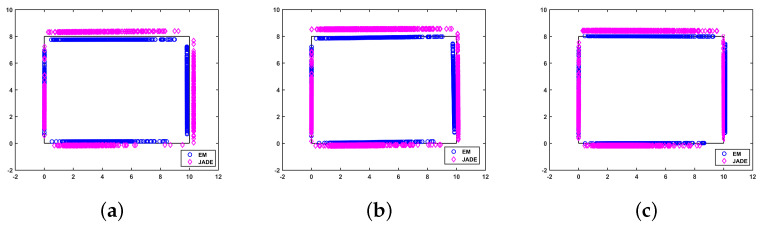
The mapping performance for different numbers of ADOA vectors: $\sigma =2^{\circ }$. (**a**) N=200; (**b**) N=500; (**c**) N=1000.

In order to evaluate the room boundary estimation capabilities, i.e., the mapping performance of the proposed method, Figure 6 and Figure 7 demonstrate the mapping performance versus the AOA measurement noise and the number of ADOA vector measurements, respectively. Figure 6 and Figure 7 show that the reconstruction of the room boundary in both the proposed algorithm and the JADE algorithm improves if the standard deviation of the ADOA measurement noise decreases or the number of samples increases, as the amount of available information in the observed ADOA measurements is larger. Moreover, the proposed algorithm reconstructs the environment more accurately than the JADE algorithm in all cases in Figure 6 and Figure 7, because the proposed method makes full use of the geometric relationship among the ADOA measurement, the MT position and the APs’ positions without any relaxation. This further confirms the merit of the proposed EM-based SLAM algorithm.

## 6. Conclusions

In this paper, we proposed a novel expectation–maximization-based simultaneous localization and mapping (SLAM) algorithm for mmW communication systems. The proposed SLAM algorithm can be readily applied to the available crowd-sourcing MPCs’ measurement data because it does not require the MPC measurements obtained at successive epochs. By fully exploiting the geometric relationship of the multipath ADOA measurements and regarding the MT positions as the latent variable of the AP positions, it employs an efficient stochastic approximation EM method to estimate both the AP positions and the MT positions and further constructs the indoor map based on the estimated AP topology. Due to the efficient processing capability of the stochastic approximation EM method and taking full advantage of the abundant spatial information in the crowd-sourcing ADOA data, the proposed method can achieve a better positioning and mapping performance than the existing geometry-based mmW SLAM method. The simulation results confirm the effectiveness of the proposed algorithm.

## Figures and Tables

**Table 1:** Comparison between EM-SLAM Methods.

	The Proposed EM-SLAM Method	The EM-SLAM Method in [23]	The EM-SLAM Method in [24]	The EM-SLAM Method in [25]
**System** **Model**	Hidden-variable models	Hidden Markov models (HMMs)	Hidden Markov models (HMMs)	Hidden Markov models (HMMs)
**E-step**	Monte Carlo approximation	Sequential Monte Carlo approximation and first-order Taylor expansion approximation	Sequential Monte Carlo approximation	EKF and first-order Taylor expansion approximation
**M-step**	Gradient descent-based optimization	Explicit maximization	Explicit maximization	Quasi-Newton minimization method
**Initial values**	unnecessary	necessary	unnecessary	necessary

## Data Availability

Not applicable.

## Abbreviations

The following abbreviations are used in this manuscript:

SLAM	Simultaneous Localization and Mapping
EM	Expectation–Maximization
mmW	Millimeter Wave
AP	Access Point
ADOA	Angle Difference of Arrival
MT	Mobile Terminal
MPCs	Multipath Components
RFS	Random Finite Set
BP	Belief Propagation
JADE	Joint Anchor and Device location Estimation
LS	Least-Squares
CLAM	Communication-driven Localization And Mapping
IMM	Interacting Multiple Model
NLOS	Non-Line of Sight
2D/3D	Two-dimension/Three-dimension

## Appendix A. Derivation for the Gradient in (14)

The gradient of the error square with respect to $a_{l}$ is derived as

(A1) $$\begin{array}{cc} \frac{\partial E_{n}^{t}}{\partial a_{l}} & =\sum_{i=1}^{L-1}\frac{\partial E_{n}^{t}}{\partial \theta_{i,i+1}\left({\hat{p}}_{n}^{t}\right)}\frac{\partial \theta_{i,i+1}\left({\hat{p}}_{n}^{t}\right)}{\partial a_{l}}\\ & =\frac{\partial E_{n}^{t}}{\partial \theta_{l-1,l}\left({\hat{p}}_{n}^{t}\right)}\frac{\partial \theta_{l-1,l}\left({\hat{p}}_{n}^{t}\right)}{\partial a_{l}}+\frac{\partial E_{n}^{t}}{\partial \theta_{l,l+1}\left({\hat{p}}_{n}^{t}\right)}\frac{\partial \theta_{l,l+1}\left({\hat{p}}_{n}^{t}\right)}{\partial a_{l}} \end{array}$$

where the second equation holds due to $\frac{\partial \theta_{i,i+1}\left({\hat{p}}_{n}^{t}\right)}{\partial a_{l}}=0$ for i≠lorl−1 from (1),

(A2) $$\begin{array}{cc} & \frac{\partial E_{n}^{t}}{\partial \theta_{l,l+1}\left({\hat{p}}_{n}^{t}\right)}=\frac{2\left(\theta_{l,l+1}\left({\hat{p}}_{n}^{t}\right)-{\tilde{\theta }}_{l,l+1}\left(p_{n}\right)\right)}{\sigma_{l,l+1}^{2}}\\ & \frac{\partial E_{n}^{t}}{\partial \theta_{l-1,l}\left({\hat{p}}_{n}^{t}\right)}=\frac{2\left(\theta_{l-1,l}\left({\hat{p}}_{n}^{t}\right)-{\tilde{\theta }}_{l-1,l}\left(p_{n}\right)\right)}{\sigma_{l-1,l}^{2}} \end{array}$$

and

(A3) $$\begin{array}{cc} & \frac{\partial \theta_{l,l+1}\left({\hat{p}}_{n}^{t}\right)}{\partial a_{l}}=-\frac{1}{\|{\hat{p}}_{n}^{t}-a_{l}{\|}_{2}}{\left[sin\left(\theta_{l}\left({\hat{p}}_{n}^{t}\right)\right),-cos\left(\theta_{l}\left({\hat{p}}_{n}^{t}\right)\right)\right]}^{T}\\ & \frac{\partial \theta_{l-1,l}\left({\hat{p}}_{n}^{t}\right)}{\partial a_{l}}=\frac{1}{\|{\hat{p}}_{n}^{t}-a_{l}{\|}_{2}}{\left[sin\left(\theta_{l}\left({\hat{p}}_{n}^{t}\right)\right),-cos\left(\theta_{l}\left({\hat{p}}_{n}^{t}\right)\right)\right]}^{T} \end{array}$$

where both equations above are derived as (26) in [6] and ${\left\|•\right\|}_{2}$ denotes the l2-norm operation.
